# Editorial: Pathophysiology of Sensitive Skin

**DOI:** 10.3389/fmed.2020.00159

**Published:** 2020-04-28

**Authors:** Laurent Misery, Howard I. Maibach

**Affiliations:** ^1^University Brest, LIEN, Brest, France; ^2^Department of Dermatology, University Hospital of Brest, Brest, France; ^3^Department of Dermatology, University of California, San Francisco, San Francisco, CA, United States

**Keywords:** sensitive skin, pathophysiology, itch, pain, reactive skin

Sensitive skin is more and more studied (1). The International Forum for the Study of Itch (IFSI), which is comparable to the International Association of the Study of Pain (IASP), initiated a special interest group on sensitive skin. This group defined sensitive skin as “a syndrome defined by the occurrence of unpleasant sensations (stinging, burning, pain, pruritus, and tingling sensations) in response to stimuli that normally should not provoke such sensations. These unpleasant sensations cannot be explained by lesions attributable to any skin disease. The skin can appear normal or be accompanied by erythema. Sensitive skin can affect all body locations, especially the face” (2). In a second position paper (3), the group concluded that new studies on the pathophysiology of sensitive skin were needed.

With the Research Topic Pathophysiology of Sensitive Skin, we aim to increase awareness for this syndrome, present new data on epidemiology and pathophysiology, and (maybe most importantly) stimulate further basic, translational and clinical research in the field.

Farage reviewed variations on the prevalence of self-declared sensitive skin according to various geographies, different gender and age groups, or various anatomic sites. In addition, she reviewed the physiological characteristics associated with this condition and factors that may contribute. Approximately 60–70% of women and 50–60% of men declared having sensitive skin. Brenaut et al. provided new data for India, with fewer amounts. Farage also reviewed data on sensitive skin in the genital area. She discussed specific conditions like higher temperature, different habits, and practices and moisture due to the semi-occlusive environment, and incontinence in some cases. The effects of menopause were also considered.

Schmelz summarized data on itch processing in the skin. Subjects reporting sensitive skin suffered from itch and pain sensations upon weak external stimuli that are not painful or itchy in the control group. An increased transduction of the external stimuli into peripheral neuronal signals followed by neuronal processing finally resulting in the perception should be involved. Because sensitive can be considered as neuropathic pruritus (NP), Huguen et al. compared the characteristics of both NP and non-neuropathic pruritus (NNP) in order to propose a new tool for the diagnosis of NP without clinical examination. A sensitivity of 76% and a specificity of 77% was obtained using a score of two criteria out of five to discriminate NP from NNP. The new tool was named NP5.

Talagas and Misery were interested in the role of keratinocytes in sensitive skin because of their role in the epidermal barrier. However, keratinocytes are probably involved in sensitive skin because they express diverse sensory receptors allowing them to have properties of transducers of pain and itch (like sensory neurons). Bataille et al. analyzed the two publications which focused on sensitive skin transcriptomics. These interesting studies showed that numerous genes may be involved in the pathogenesis and suggested pathways of sensitive skin.

Finally, Sonbol et al. studied the effects of phototherapy by light-emitting diodes (LEDs) and found impressive results on the soothing of sensitive skins, with a very good safety. Further comparative studies are needed.

Taken together, these and other recent publications emphasize that sensitive skin is real- and not an industry claim invention—suggesting that understanding the mechanisms involved—will aid the patient/consumer.

## Author Contributions

All authors listed have made a substantial, direct and intellectual contribution to the work, and approved it for publication.

## Conflict of Interest

LM has received honoraria and/or research grants from the following companies: Beiersdorf, Bioderma, Clarins, Expanscience, Galderma, Johnson&Johnson, Nestlé Skin Health, Pfizer, Pierre Fabre, Roche-Posay, Solabia, and Uriage. The remaining author declares that the research was conducted in the absence of any commercial or financial relationships that could be construed as a potential conflict of interest.
